# Transcriptome analysis of chicken intraepithelial lymphocyte natural killer cells infected with very virulent infectious bursal disease virus

**DOI:** 10.1038/s41598-020-75340-x

**Published:** 2020-10-27

**Authors:** Sook Yee Boo, Sheau Wei Tan, Noorjahan Banu Alitheen, Chai Ling Ho, Abdul Rahman Omar, Swee Keong Yeap

**Affiliations:** 1 Laboratory of Vaccines and Immunotherapeutics, Institute of Bioscience, Universiti Putra Malaysia, 43400 Serdang, Selangor Malaysia; 2 Faculty of Biotechnology and Biomolecular Sciences, Universiti Putra Malaysia, 43400 Serdang, Selangor Malaysia; 3 China-ASEAN College of Marine Sciences, Xiamen University Malaysia, Bandar Sunsuria, 43900 Sepang, Selangor Malaysia

**Keywords:** Infectious diseases, Innate immune cells, Gene expression

## Abstract

The infectious bursal disease (IBD) is an acute immunosuppressive viral disease that significantly affects the economics of the poultry industry. The IBD virus (IBDV) was known to infect B lymphocytes and activate macrophage and T lymphocytes, but there are limited studies on the impact of IBDV infection on chicken intraepithelial lymphocyte natural killer (IEL-NK) cells. This study employed an mRNA sequencing approach to investigate the early regulation of gene expression patterns in chicken IEL-NK cells after infection with very virulent IBDV strain UPM0081. A total of 12,141 genes were expressed in uninfected chicken IEL-NK cells, and most of the genes with high expression were involved in the metabolic pathway, whereas most of the low expressed genes were involved in the cytokine-cytokine receptor pathway. A total of 1,266 genes were differentially expressed (DE) at 3 day-post-infection (dpi), and these DE genes were involved in inflammation, antiviral response and interferon stimulation. The innate immune response was activated as several genes involved in inflammation, antiviral response and recruitment of NK cells to the infected area were up-regulated. This is the first study to examine the whole transcriptome profile of chicken NK cells towards IBDV infection and provides better insight into the early immune response of chicken NK cells.

## Introduction

Infectious bursal disease (IBD) is an acute immunosuppressive viral disease that affects the poultry industry with economic importance^1^. IBD is caused by the IBD virus (IBDV), a dsRNA virus belonging to the genus Avibirnavirus in the family Birnaviridae^2^. IBDV is organized into 2 segments, A and B, of about 6 kb in size. Segment A is the larger fragment and encodes the VP5, non-structural protein and another polyprotein whose post-translational cleavage gives rise to VP2, VP3 and VP4 structural proteins^3^. IBDV isolates are divided into two serotypes, 1 and 2, which both infect chickens, but the typical clinical disease is only associated with serotype 1^4^.

Infection usually occurs through the oral route. The virus replicates in avian immune cells such as the macrophages and the lymphoid cells of the duodenum, jejunum and caecum^5^. It then spreads to the bursa of Fabricius (BF) where it extensively replicates and destroys the immature B lymphocytes, thus causing a compromise in the antibody mediated immune responses in the affected chicken. The clinical severity and mortality due to IBDV depend on the presence of pre-existing maternal antibodies, age and the chicken’s genetic background^6^. Acute IBDV infections are characterized by severe clinical signs and high mortality. The incubation period is very short, about 2 to 3 days. In acute cases, the birds are exhausted, prostrated, dehydrated, suffer from aqueous diarrhoea and their feathers are ruffled. Mortality commences on the third day of infection, reaches a peak and then drops rapidly, and the surviving chickens recover a state of apparent health after 5 to 7 days^7^.

The first 3 days are the critical period for the immune response system of the chicken to respond against virus, and the innate immune response is the first layer of the defence system against a virus or bacteria. It is thus important to understand how chicken immune cells response to IBDV infection. There are number of immune cells involved in the innate immune response, including dendritic, macrophage and natural killer (NK) cells. NK cells play a key role in innate host defence against viruses. The primary physiological role of NK cells is to provide a crucial initial defence against pathological organisms during the time (from day 0 to day 5) that the adaptive immune system is still being mustered^8^. Their major function is to recognize and kill virally infected and neoplastic cells. When the ligand of NK cells interacts with cell-surface receptors, they produce several cytokines such as IFN-γ, which have an immunoregulatory role^9^.

To date, studies have been conducted to check on the expression profile of mRNA in B cells^10^, macrophages^11^, dendritic cells^12^ and embryonic fibroblast cells^13^, but there is limited understanding of the role of NK cells in IBDV infection. A study conducted by Jahromi et al.^14^ reported the expression profile for several groups of activator and suppressor NK cell receptors on the surface of 28.4^+^ IEL-NK cells using a qPCR approach and demonstrated that very virulent IBDV (vvIBDV) suppressed the activator receptors at 1-day post infection (dpi), but an overexpression of the surface activator was observed at 3 dpi. As the actual regulation of IBDV on NK cells was uncertain^15^, it is important to evaluate the response of IEL-NK cells against the vvIBDV infection, particularly at 3 dpi, to elucidate the actual role and response of IEL-NK cells in the innate immune response of chickens against this virus, which may help to establish foundations for improved prevention, including a vaccination strategy against IBDV. In this study, the whole transcriptome profiling of chicken IEL-NK cells infected by vvIBDV at 3 dpi was performed to gain insight into how such cells respond to vvIBDV.

## Results

### Isolation and enrichment of 28.4^+^ IEL-NK cells

IEL-NK cells were isolated from duodenum samples collected from uninfected chickens and chickens infected with vvIBDV at 3 dpi. The total number of IEL-NK cells was 19.48 million for control samples, 13.95 million cells for the infected chicken for 3 days. After the enrichment process using CD3 and 28.4 markers, the percentage of CD3^−^/28.4^+^ IEL-NK cells at 3 dpi were higher than in the uninfected control group, which was 42.38%.

### Measurement of viral load

The viral load titre of the samples was determined by amplifying the VP4 region in the vvIBDV strain UPM0081 using the RT-qPCR method. There was no amplification in the uninfected IEL-NK cells while the viral load was log10 of 7.10 ± 0.65 at 3 dpi.

### Identification of differentially expressed genes for infection group on day 3

The number of raw paired-end reads per sample generated from HiSeq2500 was within the range of 62 million to 69 million reads. The raw reads proceeded to adapter and quality trimming. The number of trimmed paired-end reads was within the range of 53 million to 58 million reads. The trimmed reads were mapped to chicken genome Galgal4 downloaded from the Ensembl database. The percentage of trimmed reads mapped in pairs was 72% to 81%, whereas the reads mapped in broken pairs was the range of 4.3% to 11%. The percentage of trimmed reads not mapped to the genome was within the range of 12.2% to 18.5% (Table 1).

**Table 1:** Summary of raw, trimmed and mapped reads.

dpi		Raw reads	Trimmed reads	Mapped in pair	Mapped in broken pair	Reads not mapped
0	R1	69,496,834	58,911,860	47,957,944 (81.41%)	3,765,047 (6.39%)	7,188,869 (12.2%)
	R2	67,751,426	57,247,830	44,087,180 (77%)	2,598,916 (4.54%)	10,561,734 (18.45%)
	R3	68,100,290	57,898,240	44,020,552 (76%)	4,188,992 (7.24%)	9,688,696 (16.73%)
3	R1	64,510,988	54,944,488	42,957,964 (73.55%)	2,372,524 (4.32%)	9,614,000 (17.5%)
	R2	64,643,682	55,052,202	40,490,580 (73.55%)	6,073,371 (11.03%)	8,488,251 (15.42%)
	R3	62,733,848	53,080,454	38,669,076 (72.85%)	4,879,719 (9.19%)	9,531,659 (17.96%)

Differential expression analysis was performed to find out the differential expressed (DE) genes on dpi 3 as compared to the uninfected samples. The DE gene was defined as a gene with a fold change ≥ 2 or ≤ − 2 and a False Discovery Rate (FDR) corrected *p* value < 0.05. At 3 dpi, there were 516 genes up-regulated, whereas 750 genes were down-regulated.

### GO and KEGG pathway enrichment analysis

The functions and pathways of all DE genes were analysed based on the GO and KEGG pathway. The DE genes at 3 dpi, in comparison to the uninfected samples, were enriched in a few GOs, as shown in Table 2. In terms of biological process, most of the DE genes were involved in the metabolic process, transmembrane transport, fatty acid beta-oxidation and inflammatory response. In terms of cellular components, most of the DE genes were involved in the extracellular exosome, integral component of membrane, extracellular space and peroxisome. In terms of molecular function, most of the DE genes were involved in neurotransmitter, oxidoreductase activity or haem binding.

**Table 2:** Top five enriched GOs for DE genes at 3 dpi.

Gene ontology	Term ID	Description	*p* value
Biological process	GO:0,008,152	Metabolic process	4.47E−04
	GO:0,055,085	Transmembrane transport	5.35E−04
	GO:0,033,539	Fatty acid beta-oxidation using acyl-CoA dehydrogenase	0.0035
	GO:0,006,954	Inflammatory response	0.0044
	GO:0,005,975	Carbohydrate metabolic process	0.0105
Cellular component	GO:0,070,062	Extracellular exosome	2.05E−05
	GO:0,016,021	Integral component of membrane	1.97E−04
	GO:0,005,615	Extracellular space	5.79E−04
	GO:0,005,777	Peroxisome	0.0015
	GO:0,009,986	Cell surface	0.0063
Molecular function	GO:0,005,328	Neurotransmitter:sodium symporter activity	0.0020
	GO:0,016,491	Oxidoreductase activity	0.0055
	GO:0,020,037	Heme binding	0.0086
	GO:0,004,252	Serine-type endopeptidase activity	0.0098
	GO:0,005,506	Iron ion binding	0.0135

**Figure 1:**
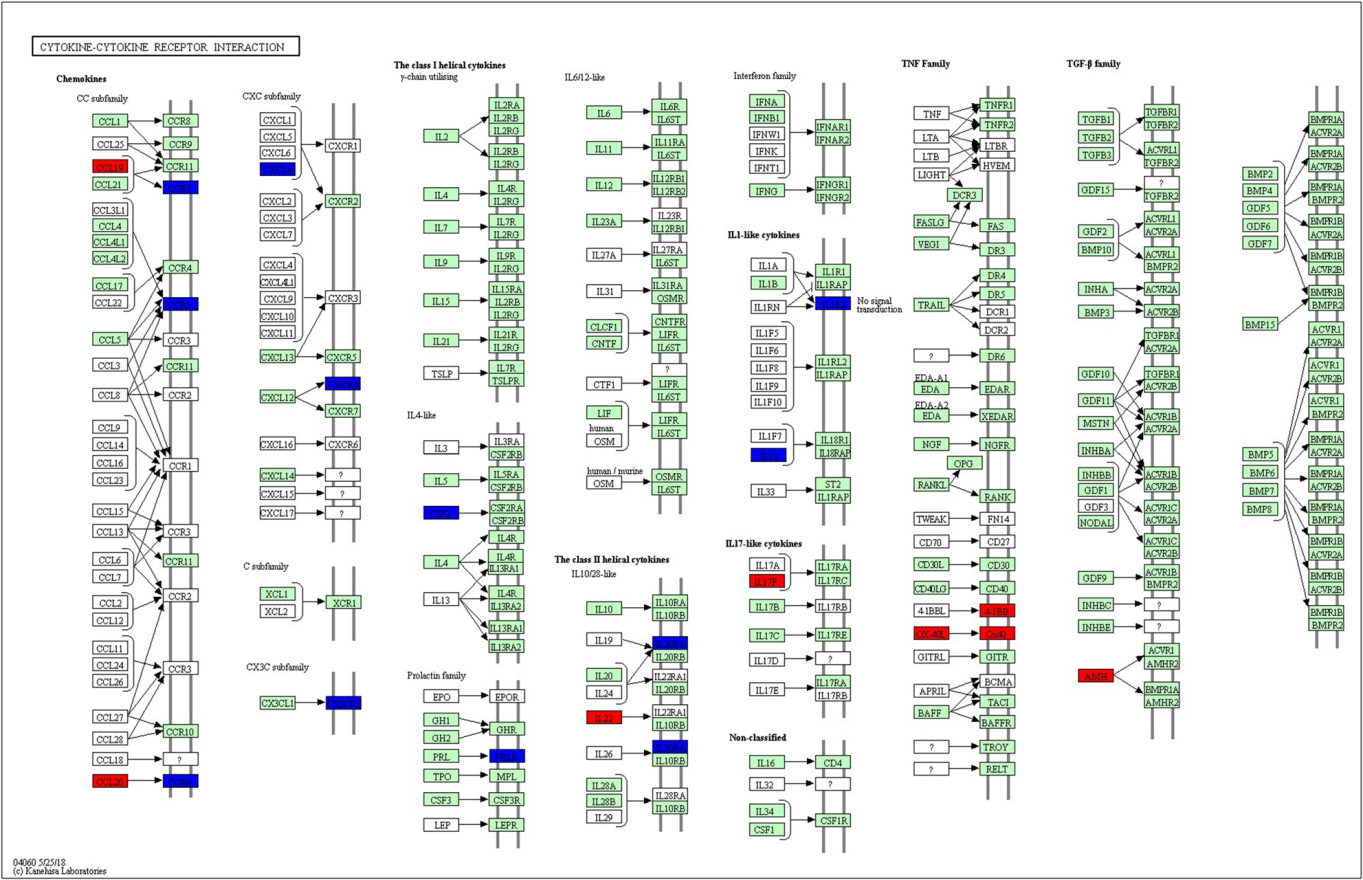

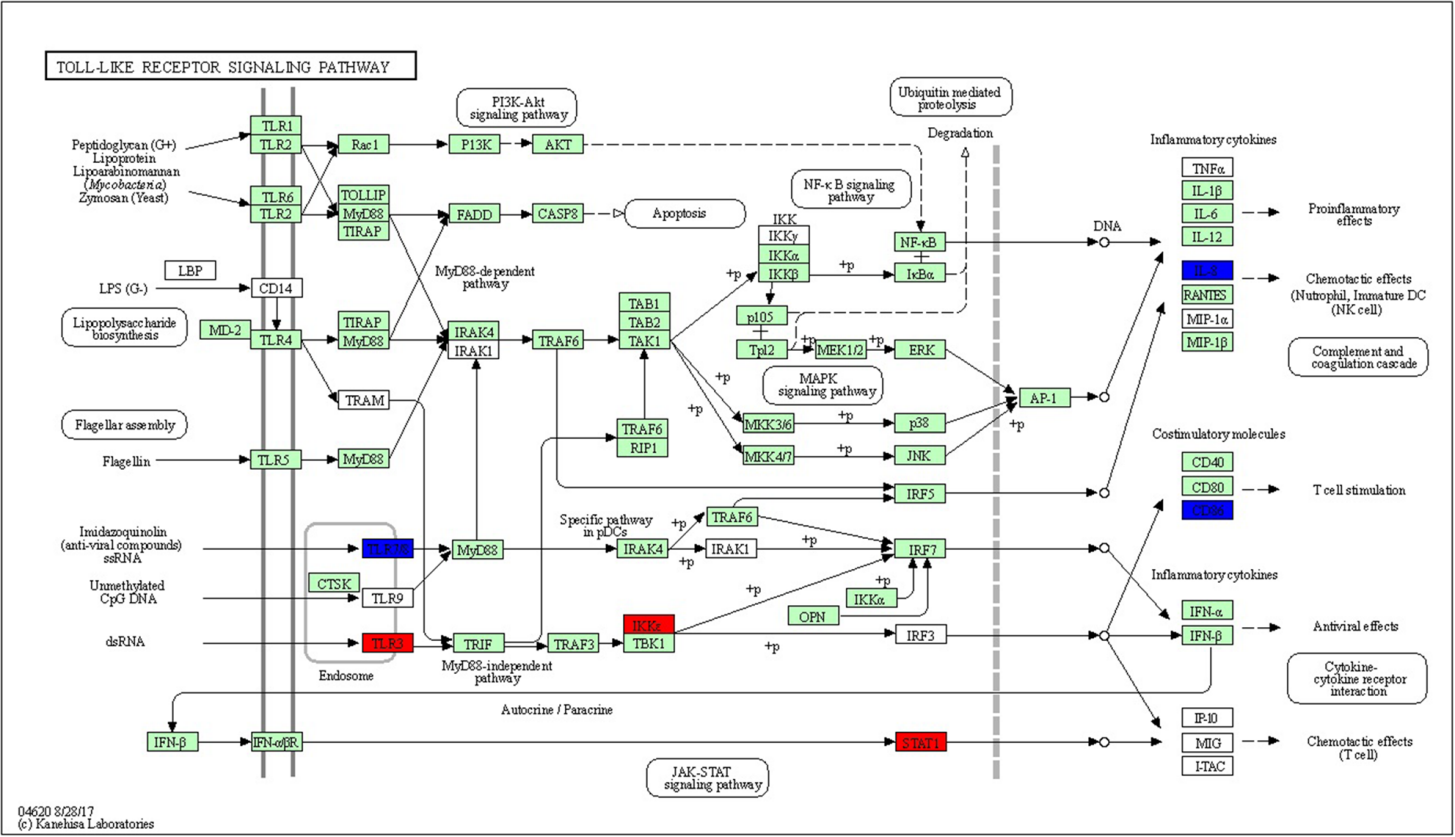

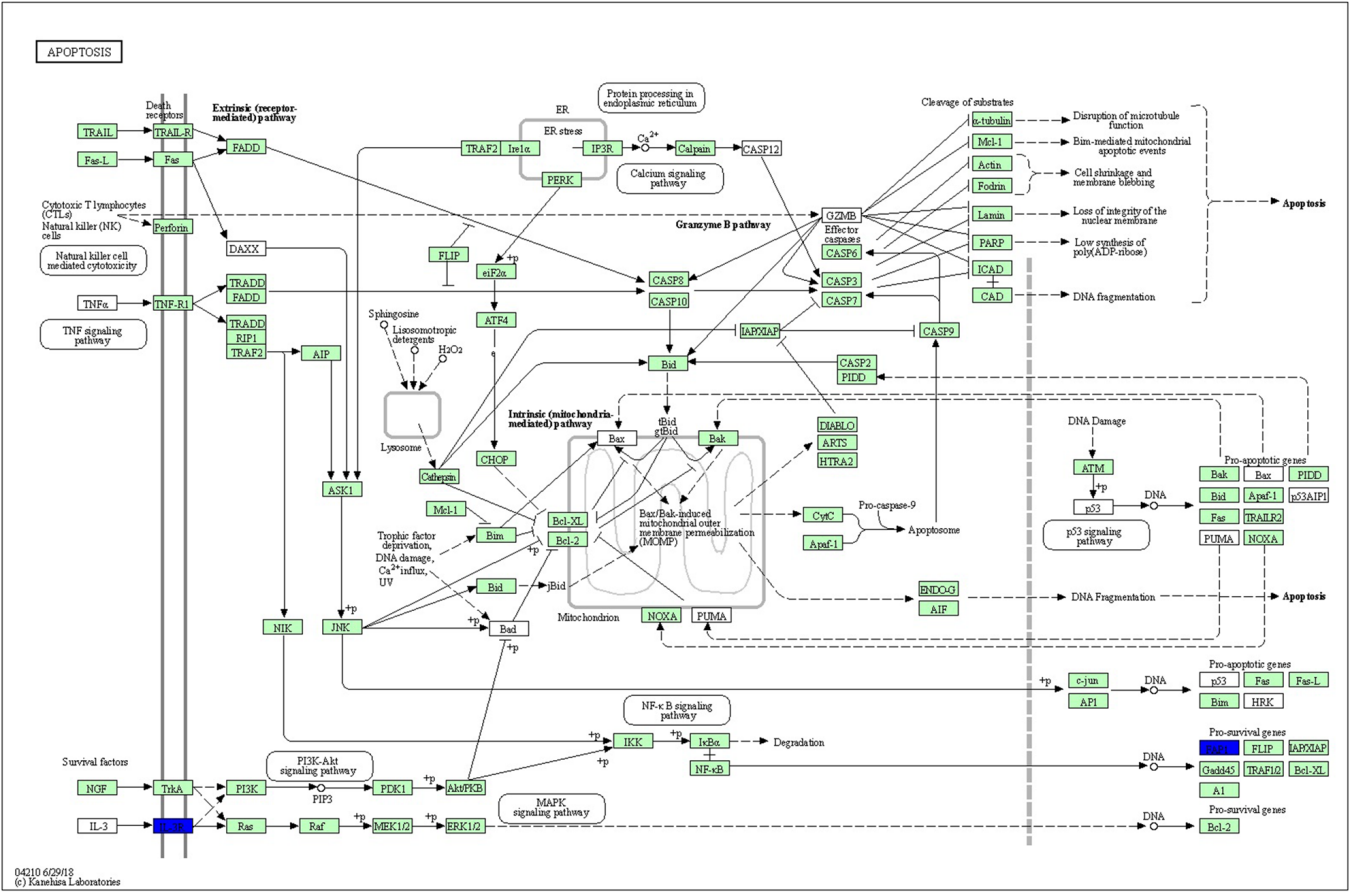
KEGG pathway enrichment analysis of the host response towards vvIBDV infection. These KEGG pathways were generated using DE genes from IEL-NK cells obtained from RNA-Seq. (**a**) Cytokine-cytokine receptor interaction pathway. (**b**) Toll-like receptor signalling pathway. (**c**) Apoptosis pathway. The down-regulated DE genes are highlighted in blue and up-regulated DE genes are highlighted in red. Pathways were adopted from KEGG pathway database^59^. The green and white coloured boxes are default colours generated by the software, depicting genes that were not the DE on RNA-Seq, and unidentified genes in the organism-specific pathway, respectively.

KEGG Pathway enrichment analysis showed that most of the DE genes at 3 dpi were clustered into metabolic pathways or pathways of valine, leucine and isoleucine degradation, biosynthesis of antibiotics and tryptophan metabolism (Table 3). Meanwhile, a total of 20 DE genes were involved in the cytokine-cytokine receptor interaction pathway (Fig. 1a). Out of the 20 DE genes, 12 genes were down-regulated and 8 genes were up-regulated after infection by vvIBDV at 1 dpi and 3 dpi. Moreover, 7 DE genes (4 down-regulated and 3 upregulated) were involved in the Toll-Like Receptor (TLR) signalling pathway (Fig. 1b). There were only 2 DE genes involved in the apoptosis pathway (Fig. 1c).

**Table 3:** Top 10 enriched KEGG pathways for DE genes at 3 dpi.

ID	Description	Number of genes	*p* value
gga01100	Metabolic pathways	146	2.51E−12
gga00280	Valine, leucine and isoleucine degradation	17	1.76E−07
gga01130	Biosynthesis of antibiotics	34	1.05E−05
gga00380	Tryptophan metabolism	13	4.44E−05
gga01200	Carbon metabolism	21	8.53E−05
gga00561	Glycerolipid metabolism	13	0.0010
gga00071	Fatty acid degradation	10	0.0011
gga00040	Pentose and glucuronate interconversions	7	0.0018
gga00010	Glycolysis / Gluconeogenesis	12	0.0025
gga00640	Propanoate metabolism	8	0.0033

### Apoptosis and inflammatory

Some of the genes related to apoptosis and inflammation were differentially expressed after vvIBDV infection. Of the apoptosis-related genes, *CASP1* was up-regulated at 3 dpi with a fold change of 2.14. All of the inflammation-related genes up-regulated at 3 dpi and these genes are *CCL19, CCL20, IL17A, IL22, LIPA, P2RX7, tac1, TLR3* and *TNFSF4*.

### Cytokine, chemokines and interferon stimulation

Most of the genes for chemokines/chemokine receptors (*CCR2, CCR6, CCR7, CSF2RB, CX3CR1* and *CXCR4*) and cytokines/cytokines receptors (*IL1R2, IL8, IL18, IL20RA* and *IL22RA2*) were down-regulated after infection by vvIBDV at 3 dpi. However, the genes for *CCL19* and *CCL20* were found to be up-regulated with a fold change of 2.53 and 2.09 while the genes for IL22 cytokines were up-regulated with a fold change of 3.76 at 3 dpi. Other than the genes for chemokines and cytokines, several genes for TLRs were also differentially expressed as a result of the vvIBDV infection. *TLR7* was down-regulated, while *TLR3* was the only TLR gene up-regulated at 3 dpi, with a fold change of 2.22.

The IFN-stimulated genes (ISGs) are involved in antiviral defence. Some of the ISGs genes such as *IFIT5, IFITM5, MX1, RSAD2* and *SAMHD1* were up-regulated at 3 dpi. Among these five genes, *RSAD2* had the highest fold change of 5.26. Additionally, the tumour necrosis factor related genes, *TNFRSF9* and *TNFSF4*, were up-regulated at 3 dpi with a fold change of 2.28 and 4.86, respectively. The list of DE genes related to DNA replication, cell cycle, inflammation, cytokine, chemokine and interferon stimulation is summarized in supplementary file 1.

### Avian NK cell surface receptors

The gene expression level for avian NK cells surface receptors such as *CD69*, *CHIR-AB1*, *B-Lec* and *B-NK* was identified through the RNA-Seq and RT-qPCR results. The expression of *CD69, B-NK* and *B-Lec* genes were up-regulated at 3 dpi compared to the uninfected samples. However, the gene expression for the bifunctional marker, *CHIR-AB1*, was similar to uninfected samples (Table 4).

**Table 4:** Fold change of NK cell receptors infected with ELD_50_ 10^3^ of vvIBDV strain UPM0081.

Gene	3 dpi
*CD69* (activator)	3.0^a^
*CHIR-AB1* (bifunctional marker)	1.3^a^
*B-Lec* (activator)	2.5^b^
*B-NK* (repressor)	4.67^b^

^a^According to RT-qPCR result.

^b^According to RNA-Seq result.

### RNA-Seq result validated using NanoString technology

**Figure 2:**
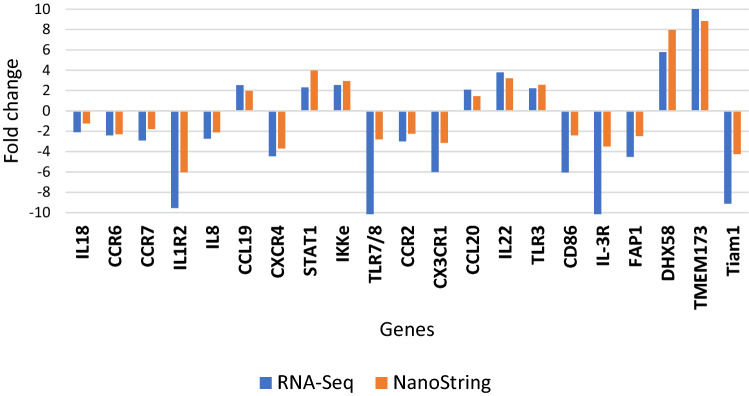
The fold change of DE genes analysed with RNA-Seq and NanoString.

NanoString Technology was used to validate the expression level of 21 DE genes involved in 6 pathways related to the innate immune response against vvIBDV infection (Fig. 2). From the 21 DE genes, 19 genes showed similar expression profile to the RNA-Seq results. Two genes could not be compared to the RNA-Seq results, as the expression levels of these two genes (*CX3CL1* and *CD86*) were not significant due to the high p-value (> 0.05).

## Discussion

In model organisms such as humans, rats and mice, NK cells have been well characterized^16,17^. The number of studies focusing on avian NK cells is, however, very limited, mainly due to the unavailability of specific mAbs against avian NK cells. The avian NK cell–specific 28.4 mAb was discovered by Göbel et al.^18^ and this mAb can be used to isolate 28.4^+^ IEL-NK cells. Further study from Gobel’s group found that the 28.4^+^ cells are predominantly present in the chicken duodenum, and these 28.4^+^ cells play important roles against pathogenic organisms. In this study, the CD3^−^/28.4^+^ IEL-NK cells were isolated from uninfected chickens and chickens infected with vvIBDV for 3 days.

To confirm if the IEL-NK cells were activated or inhibited by vvIBDV at 3 dpi, the expression of chicken NK cell receptors, including *CD69, B-Lec, CHIR-AB1* and *B-NK*, were examined. CD69 is one of the cell surface receptors expressed on human and mouse haematopoietic leukocytes^19^. CD69 acts as the activating receptor of NK cells and the expression of *CD69* is low in unstimulated human NK cells^20^. B-Lec also acts as an activating receptor on human NK cells that contain an endocytosis motif^21^. CHIR-AB1 is a receptor that combines inhibitory and activating features. It is found expressed on NK cells that are present in the IEL, peripheral blood and spleen^22^. Meanwhile, B-NK is an inhibitory receptor on the surface of NK cells with an ITIM signalling motif^21^. Pathogenicity of other types of virus, such as avian influenza virus and Newcastle disease virus (NDV), has influenced the activation of duodenum and lung NK cells^23,24^. Jansen et al.^24^ has observed that there is elevated activation of chicken lung NK cells following infection with a low pathogenic avian influenza virus (LPAI), H9N2*,* whereas there is decreased activation for lung NK cells after infection with a high pathogenic avian influenza virus, H5N1. According to the study by Abdolmaleki et al.^23^, the expression of chicken NK cell receptors such as *CD69, B-Lec, NK-lysin* and *IFN-γ* were down-regulated after infection with velogenic NDV strains, whereas chickens infected with a vaccine strain of NDV showed minor effects on both the expression of their surface receptors and the total population of CD3^−^/28.4^+^ IEL-NK cells. A previous study by Jahromi et al.^14^ has reported that, when infected with vvIBDV, all the activator markers (i.e. *CD69* and *B-Lec*) and the bi-functional marker *CHIR-AB1* were upregulated, while the inhibitory marker B-NK was not significantly changed. In this study, expression of *CD69* and *B-Lec* had the same trend as the previous report, while the bi-functional marker *CHIR-AB1* was not significantly regulated. Overexpression of *CD69* and *B-Lec* suggests that the IEL-NK cells were activated by the vvIBDV at 3 dpi.

**Figure 3:**
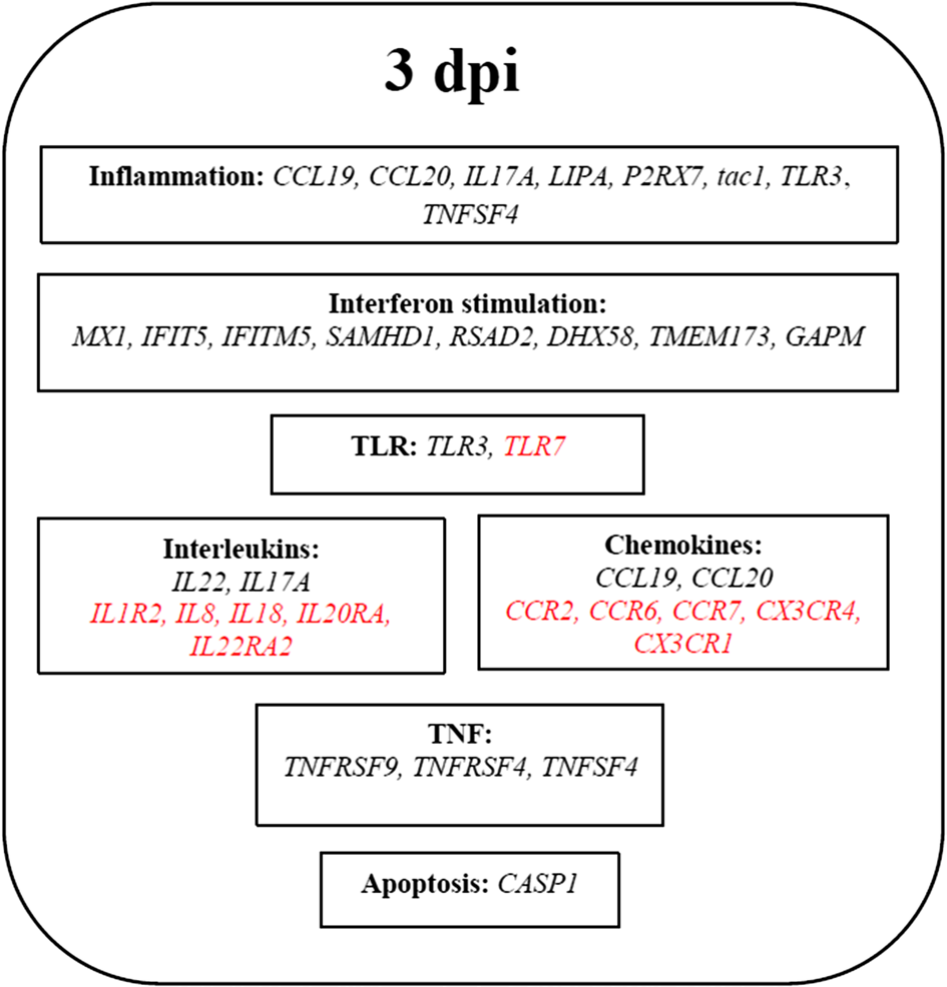
Schematic diagram showing the gene expression profile of DE genes at 3 dpi. The genes highlighted in red indicate down-regulated genes.

The DE genes in the response of CD3^−^/28.4^+^ IEL-NK to vvIBDV infection, which mainly involved the Toll signalling pathway, inflammatory response, interferon stimulation and cytokine and chemokines pathways, have supported the indication of IEL-NK cell activation as proposed by the upregulation of the activation markers. The DE genes important for the innate immune response towards vvIBDV are summarized in Fig. 3.

Jahromi et al.^14^ have reported the viral load of CD3^−^/28.4^+^ IEL-NK cells isolated from a chicken infected by ELD_50_ 10^5.4^ of the vvIBDV strain UPM0081, but have concluded that vvIBDV entered the cells but was not able to replicate effectively. Detection of a low viral copy number in the IEL-NK cells in this study was similar to the finding by Jahromi et al. ^14^. When the host is infected by a virus, the NOD-like receptors or TLRs on the surface of immune cells involved in the innate immune response rapidly sense and recognize the conserved features of the virus entering the host. These receptors are differentially expressed among different immune cells, which is responsible for the pro and anti-inflammatory responses. The innate immune system recognizes a specific pathogen by replying on germline-encoded PRRs that have evolved to detect Pathogen-Associated Molecular Patterns (PAMPs), which are a component of foreign pathogens^25,26^. TLR3 is a receptor that recognizes dsRNA and induces antiviral responses by triggering the production of inflammatory cytokines and type I interferon. The TLR recognition mechanism was clarified by the structural analysis of human TLR3 ectodomain bound to dsRNA^27,28^. TLR3 is mainly expressed in the surface of the endosome of immune cells and fibroblasts for dsRNA virus recognition^27,28^. The expression level of *TLR3* varies among different immune cells, but *TLR3* was upregulated in most of the studies related to IBDV infection^29,30^ and similar results were found in this study. The presence of viral particles in the IEL-NK cells may contributed to the upregulation of *TLR3*, which was reported in this study and by Jahromi et al.^14^. Sensing of the dsRNA virus by TLR3 induced the production of IFN and initiates signalling pathways, such as NFkB and the MAPK cascades, which resulted in the expression of pro-inflammatory mediators^31^.

Inflammation is a protective response towards any harmful stimuli, such as virus or bacterial infection and physical injury, by triggering the migration of immune cells to the infected or injured area. In this study, some of the inflammatory related genes (*CCL19*, *CCL20*, *IL17A*, *IL22*, *LIPA*, *P2X7*, *tac1*, *TLR3* and *TNFSF4*) were upregulated at 3 dpi (Additional file 1). Upregulation of chemokines, *CCL19* and *CCL20*, were observed, which help in the migration of NK cells to the infected area. Wang et al.^32^ had the same finding, where the mRNA levels of *CCL19* were upregulated after infection with vvIBDV. Their data suggest that CCL19 acts as a chicken peripheral white blood cells (PWBC) chemotactic factor and facilitates the infiltration of PWBC into the bursa after IBDV infection^33^. Interleukins such as *IL17A* and *IL22*, which were upregulated at 3 dpi, are important to maintain mucosal immunity against virus infection and include induction of antiviral proteins, recruitment of neutrophils to infected areas and enhancement of mucosal barrier repair. IL22 was identified to be regulating mucosal epithelial cell function, maintaining barrier integrity and protecting against bacterial and viral infection in the gut and lung^34–36^.

ISGs are documented to be involved in antiviral defence by targeting any step in a virus life cycle to limit virus replication and enhance IFN production^37^. ISGs such as *MX1, IFIT5, IFITM5, SAMHD1, RSAD2*, *DHX58, TMEM173* and *GAPM* were upregulated at 3 dpi (Additional file 1). These ISGs have been suggested to play important role in IBDV antiviral activities. MX1 is a hydrolase enzyme with antiviral characteristics which are induced by interferon I and III. It blocks the replication and transcription of the virus to protect the host from virus infection^38^. IFN-induced proteins with tetratricopeptide repeats (IFITs) are a family of antiviral proteins which induces IFN signalling. The IFIT family consists of 4 canonical human members (IFIT1, IFIT2, IFIT3 and IFIT5) which are induced upon stimulation with IFN and virus infection^39^. Zhang et al.^40^ has reported that the expression of mRNA and protein for IFIT5 was increased after detecting the presence of RNA virus in the host body, which indicates that it plays a role in the innate immune response to virus infection. Sterile alpha motif and HD-domain-containing protein 1 (SAMHD1) blocks the replication of retrovirus and certain DNA viruses by reducing the intracellular pool of dNTP^41–43^. The expression of radical S-adenosyl methionine domain-containing protein 2 (RSAD2) was up-regulated as part of the antiviral defence response against viruses such as bovine respiratory syncytial virus (BRSV) and hepatitis C virus (HCV) ^44,45^.

TNFRSF4 (OX40) and TNFSF4 (OX40L) were upregulated at 3 dpi compared with the uninfected samples. TNFRSF4 is a receptor for TNFSF4, and these two genes play an important role for NK cell proliferation. TNFSF4 was expressed in human NK cells after activation by ligation; the activated NK receptors then signal through the ITAM-bearing DAP12 adapter proteins. It was reported that TNFSF4 was up-regulated in a duck infected by avian influenza virus reservoir species^46^ and human dendritic cells infected with Ebola virus^47^. These findings suggest that TNFSF4 was being expressed in human NK cells and the expression level increased after infection by the virus. TNFRSF4 previously was known only as expressed in T lymphocytes^48^. However, the recent studies reported by Pollmann et al.^49^ have shown that TNFRSF4 was not expressed on naïve NK cells, but its expression increased after NK cells were activated by monocyte-derived cells stimulated by HCV. Monocyte-derived cells and the OX40/OX40L axis are triggered by the cell-to-cell contact mediated mechanism of NK cell activation and proliferated in response to HCV. This study has thus proposed that vvIBDV promoted the expression of TNFRSF4 in IEL-NK.

The percentage of CD3^−^/28.4^+^ IEL-NK cells was increased, but the total number of IEL-NK cells per vvIBDV in infected chicken was reduced compared to the healthy control chicken at 3 dpi^15^. The same phenomena were observed by Jahromi et al.^14^, where a higher percentage but lower total number of CD3^−^/28.4^+^ IEL-NK cells was recorded at 3 dpi compared to the uninfected control group. The reduced number of the total IEL-NK cells per chicken was mainly due to the drastic reduction of IEL cells in the infected chicken. The above evidence shows that NK cells were activated at 3 dpi to suppress the virus activity after sensing the dsRNA of vvIBDV, which may contribute to the higher percentage of the IEL-NK cells in the vvIBDV infected chicken. However, immunosuppressive and apoptosis related gene expressions were also observed in the IEL-NK cells infected with vvIBDV. For example, *IL18* was down-regulated at 1 and 3 dpi. IL18 plays a key role in inducing IFN-γ production, the proliferation of activated T lymphocytes and activation of NK cells^50^. Both IL12 and IL18 stimulate the production of IFN-γ by NK cells during viral infection^51,52^. Down-regulation of IL18 suggests that the NK cells were being deactivated and the innate immune response was suppressed. Although expression of *IL-22* was upregulated, expression of its receptor *IL22RA2* was also found to be downregulated. In addition, some of the chemokine receptors such as *CX3CR1, CX3CR4, CCR2, CCR6* and *CCR7* were down-regulated after IBDV infection. The chemokines CCR2 and CX3CR1 regulate NK cell recruitment upon inflammation^53^. Furthermore, downregulation of these proinflammatory cytokines was also supported by the concurrent detection of upregulation of the IEL-NK cells inhibitory marker, *B-NK*. Moreover, CASP1 was found upregulated at 3 dpi. Caspases are a family of conserved cysteine proteases that play important roles in regulating apoptosis^54,55^. Severe inflammation may have contributed to this effect^56^ and can be the factor contributing to the lower total number of IEL cells in the vvIBDV infected chicken than in the healthy control chicken.

In conclusion, innate immunity represented by the IEL-NK cells was activated as several genes involved in inflammation, antiviral response and recruitment of NK cells to the infected area were up-regulated. However, concurrent immune suppression also occurred, particularly in the downregulation of T cell activating cytokines and several chemokines, which may limit the antiviral effect of the IEL-NK cells. To connect the role of IEL-NK cells with the gut adaptive immunity of chicken after vvIBDV infection and elucidate the complete gut immune response of chickens against infection, further study is required.

## Materials and methods

### Ethical considerations

Based on the reference number UPM/IACUC/AUP-R051/2014, all animal experiments were approved by the local animal care authority and pathogenicity study by the Institutional Animal Care and Use Committee (IACUC), Faculty of Veterinary Medicine, Universiti Putra Malaysia (UPM), following the ethical guidelines for the care and use of lab animals by the committee.

### Chickens and viruses

Nine-day-old SPF embryonated chicken eggs were purchased from Veterinary Research Institute, Ipoh, Perak, and incubated under sterile conditions at the Laboratory of Vaccines and Immunotherapeutics, Institute of Bioscience (IBS), UPM. Once hatched, the chickens were transferred to a Biosafety Level-2 (BSL-2) animal house facility, where they were fed with pelleted feed and supplied with water ad libitum. The vvIBDV strain UPM0081 was kindly provided by Prof Dr Abdul Rahman Omar from IBS, UPM, Malaysia.

At 4 weeks of age, 48 SPF chickens were randomly divided into two groups (i.e. 24 chickens in each group): the control group (without viral infection/healthy group) and the chickens infected with vvIBDV for 3 days. Chickens from the infection groups were challenged with the vvIBDV strain UPM0081 stock at a dosage of 10^3^ EID_50_ in a volume of 0.1 ml through eye-nose drops. The chickens in the control group were treated with 1 × Phosphate-Buffered Saline (PBS) (Sigma, St Louis, MO, USA), pH 7.4. All of the chickens were housed under a 12-h dark–light cycle and sterilised tap water and a standard pellet-diet were provided throughout the study. At 3 days post-infection (dpi), all chickens from each group were sacrificed under anaesthesia using carbon dioxide for duodenum collection. The duodenal loops were harvested and submerged in sterile-cold Roswell Park Memorial Institute (RPMI) 1640 medium (R6504, Sigma, St Louis, MO, USA) before IEL isolation was carried out.

### CD3^−^28.4^+^IEL-NK cell isolation

The isolation of IEL-NK cells from the duodenum was carried out as described by Jahromi^14^. About 2 × 10^8^ isolated IEL cells were resuspended in 100 µl PBS-BSA-EDTA buffer and 10 μl of CD3 Phycoerythrin (PE) mAb (8200-09, Southern Biotech, Birmingham, Alabama, USA) was added. The CD3^−^ cells that passed through the MACS BS column (Miltenyi Biotec, Bergisch Gladbach, Germany) were collected and then labelled with 28.4 mAb (kindly provided by Professor Thomas Göbel, Germany). The purity and quantity of the CD3^−^28.4^+^ IEL-NK cells were measured using a flow cytometer (BD FACSCalibur, San Jose, CA, USA) as presented by Boo et al.^57^. All CD3^−^28.4^+^ IEL-NK cells obtained from eight chickens were pooled together as one biological replicate, giving a total of three biological replicates per group.

### RNA extraction and mRNA isolation

Total RNA was extracted from IEL-NK cells using the Trizol method following the manufacturer’s instructions and checked for RNA integrity number to inspect RNA integrity by Agilent 2100 Bioanalyzer (Agilent technologies, Santa Clara, CA, US). The mRNA was isolated from total RNA using the NEBNext Poly(A) mRNA Magnetic Isolation Module (E7490S, NEB, Ipswich, MA, USA) following the manufacturer’s instructions. The mRNA samples were used for NanoString, RNA-Seq and RT-qPCR assay.

### cDNA synthesis and viral load determination

The extracted RNA was reverse transcribed into cDNA using the NEXscript cDNA synthesis kit (Geneslabs, Gyeonggi-do, Korea). Briefly, 4 µl of 5 × RT buffer, 1 µl of RTase enzyme and up to 1 µg of RNA sample were mixed together. The total volume was adjusted to 20 µl using nuclease free water. The mixture was placed in a thermocycler at 50 °C for 60 min followed by 95 °C for 5 min to inactivate the reverse transcriptase enzyme. The final cDNA product was used for the viral load test. SYBR green-based real-time PCR assay was employed to quantify the viral load of IBDV in CD^−^/28.4^+^ IEL-NK cells at various time points following vvIBDV infection. The assay uses primers that target the VP4 gene of IBDV^58^. The PCR reaction was performed in a CFX96 Real Time System (BioRad, Hercules, California, USA) as follows: 95 °C for 3 min followed by 40 cycles of denaturation at 95 °C for 30 s, annealing at 60 °C for 20 s, extension at 72 °C for 40 s and melt curve analysis was carried out at 70 °C to 95 °C with increments of 0.5 °C every 5 s per step.

### Library preparation and sequencing

The library preparation was conducted using the ScriptSeq v2 library prep kit (Illumina, San Diego, CA, USA) following the manufacturer’s instruction with modifications. At least 50 ng of mRNA was used for the library preparation. The quality and quantity of the final library were being checked using Qubit (Thermo Fisher Scientific, Waltham, MA, USA), qPCR by CFX96 Real Time System (BioRad, Hercules, California, USA) and Agilent 2100 Bioanalyzer (Agilent technologies, Santa Clara, CA, US). The final libraries were loaded into the Illumina cBot system for cluster generation, followed by sequencing (2 × 101 cycles) in the Illumina HiSeq2500 according to standard protocols. The data for each sample were not less than 60 million reads and the percentage of Q30 was more than 90%.

### Differential expression analysis

After the sequencing was complete, the obtained BCL files were transformed into FASTQ files using the BCL2FASTQ conversion software. FastQC was used to check the quality of the reads and adapter dimer contamination. The raw reads were loaded into the CLC Genomics Workbench and cleaned to remove the low-quality reads, including adapter and sequences shorter than 50 nt with Q < 30 at 3′ end. The clean reads were mapped to the chicken genome (GalGal4, Ensembl release 85). The differentially expressed (DE) genes were identified with fold changes ≥ 2 and FDR corrected *p* value < 0.05. The raw sequencing data and processed data files have been deposited in the Gene Expression Omnibus (GEO) at the NCBI under accession number GSE123920.

### Functional annotation and pathway enrichment analysis

The DE genes were analysed using the web-based tools in DAVID to identify enriched GO terms and KEGG pathways, to group functionally related genes and to cluster the annotation terms.

### NanoString nCounter assay

NanoString nCounter Elements probes for the 21 DE genes stated in Table 5 were designed by the NanoString Bioinformatics team (NanoString Technologies, Seattle, WA, USA) and synthesized by Integrated DNA Technologies (IDT) in Singapore. The list of genes was selected from key pathways related to innate immune response such as cytokine-cytokine receptor interaction, TLR signalling, apoptosis and other pathways. The gene expression of 21 genes was measured using a multiplexed hybridization assay and specific fluorescent barcode probes with no amplification step; 15 reference genes were included in the panel for normalization.

**Table 5:** DE genes being validated using NanoString technology.

No	Pathways	DE genes
1	Cytokine-cytokine receptor interaction	*IL18, IL22, IL1R2, IL8 (CXCLi2), CCL19, CCL20, CCR2, CCR6, CCR7, CXCR4-201, CX3CR1*
2	Toll-like receptor signaling pathway	*TLR3, TLR7/8, STAT1, IKKe, CD86*
3	Apoptosis	*IL-3R, FAP1*
4	RIG-Like receptor signaling pathway	*LGP2, MITA*
5	Chemokine signaling pathway	*Tiam1*

Briefly, total RNA was diluted in nuclease free water to 40 ng/µl, making a final assay concentration of 200 ng. Samples were incubated 16–21 h at 67 °C per the manufacturer’s standard protocol to ensure hybridization with reporter and capture probes. After hybridization, the samples were processed in the Prep Station (NanoString Technologies, Seattle, WA, USA) and counted in the digital analyser (NanoString Technologies, Seattle, WA, USA); nSolver Analysis Software version 4.0 (NanoString Technologies, Seattle, WA, USA) was used to perform data quality checks, spike-in-control normalization and reference gene normalization. Datasets from triplicates were grouped and fold change estimates were calculated by building ratios between the infected group and the control group at 3 dpi.

## Supplementary information

Supplementary Information.

## Data Availability

The authors declare that all the data in this manuscript are available.
