# Supplementary material for: Transcriptome analysis of chicken intraepithelial lymphocyte natural killer cells infected with very virulent infectious bursal disease virus
Source: Sci Rep. 2020 Oct 27;10:18348. doi: 10.1038/s41598-020-75340-x (PMC7591896; doi:10.1038/s41598-020-75340-x)
Supplement: Supplementary file 1 — Supplementary Information. [file 41598_2020_75340_MOESM1_ESM.docx]

**Transcriptome Analysis of Chicken Intraepithelial Lymphocyte Natural Killer Cells Infected with Very Virulent Infectious Bursal Disease Virus**

Sook Yee Boo^1^, Sheau Wei Tan^1^, Noorjahan Banu Alitheen^2^, Chai Ling Ho^2^, Abdul Rahman Omar^1^, Swee Keong Yeap^1,3,*^

^1^Laboratory of Vaccines and Immunotherapeutics, Institute of Bioscience, Universiti Putra Malaysia, 43400 Serdang, Selangor, Malaysia

^2^Faculty of Biotechnology and Biomolecular Sciences, Universiti Putra Malaysia, 43400 Serdang, Selangor, Malaysia

^3^China-ASEAN College of Marine Sciences, Xiamen University Malaysia, Bandar Sunsuria, 43900 Sepang, Selangor, Malaysia.

* Correspondence should be addressed to Yeap Swee Keong; skyeap@xmu.edu.my

**Supplementary file 1.** **List of DE interleukins, chemokines and DE genes related to inflammation and interferon stimulation**

List of DE interleukins at 3 dpi

| Feature ID | Fold change | FDR p-value correction |
| --- | --- | --- |
| IL17A | 2.91 | 0.01 |
| IL18 | -2.09 | 0.02 |
| IL1R2 | -9.56 | 7.68E-3 |
| IL20RA | -2.77 | 0.01 |
| IL22 | 3.79 | 9.45E-3 |
| IL22RA2 | -8.67 | 0.04 |
| IL8 | -2.74 | 0.03 |

List of DE chemokines at 3 dpi

| Feature ID | Fold change | FDR p-value correction |
| --- | --- | --- |
| CCL19 | 2.53 | 0.03 |
| CCL20 | 2.09 | 5.06E-3 |
| CCR2 | -3 | 0.02 |
| CCR6 | -2.41 | 0.02 |
| CCR7 | -2.91 | 0.02 |
| CX3CR1 | -6 | 0.02 |
| CXCR4 | -4.47 | 4.81E-3 |

List of DE genes involved in inflammation at 3 dpi.

| Feature ID | Fold change | FDR p-value correction |
| --- | --- | --- |
| ANXA1 | -5 | 0.04 |
| C3AR1 | -3.61 | 0.03 |
| CCL19 | 2.53 | 0.03 |
| CCL20 | 2.09 | 5.06E-3 |
| CCR2 | -3 | 0.02 |
| CCR7 | -2.91 | 0.02 |
| CD180 | -6.25 | 0.04 |
| IL17A | 2.91 | 0.01 |
| IL18 | -2.09 | 0.02 |
| IL22 | 3.79 | 9.45E-3 |
| KIT | -6.62 | 5.12E-3 |
| LIPA | 2.74 | 8.04E-3 |
| LOC378902 | -2.68 | 0.01 |
| LY86 | -37 | 0.01 |
| P2RX7 | 2.05 | 9.32E-3 |
| SELP | -5.71 | 0.03 |
| tac1 | 2.7 | 0.04 |
| TLR3 | 2.22 | 0.02 |
| TLR7 | -35.14 | 0.01 |
| TNFSF4 | 4.86 | 0.03 |

List of DE genes related to the interferon stimulation at 3 dpi.

| Feature ID | Fold change | FDR p-value correction |
| --- | --- | --- |
| BNIP3 | -2.1 | 0.02 |
| BNIP3L | -2.21 | 0.03 |
| GPAM | -2 | 0.05 |
| HYAL2 | 2.37 | 0.04 |
| IFITM5 | 4.65 | 0.03 |
| IFIT5 | 3.96 | 7.73E-3 |
| MX1 | 4.86 | 9.26E-4 |
| RSAD2 | 5.26 | 3.18E-3 |
| SAMHD1 | 2.76 | 3.89E-3 |
| TLR3 | 2.22 | 0.02 |
| TLR7 | -35.14 | 0.01 |
| TMEM173 | 14.14 | 0.03 |
